# Water-pipe smoking and serum testosterone levels in adult males in Qatar

**DOI:** 10.18332/tid/99572

**Published:** 2019-03-13

**Authors:** Mahmoud Y. Haik, Anas A. Ashour, Yaman F. M. Alahmad, Fajer A. Al-Ishaq, Mona M. Saad, Maha M. Hussein, Reem S. Mubarak, Wafaa A. Mohamed, Ala-Eddin Al Moustafa

**Affiliations:** 1 College of Medicine, Qatar University, Doha, Qatar; 2 Biomedical Research Centre, Qatar University, Doha, Qatar

**Keywords:** water-pipe smoking, free testosterone, sex hormone binding globulin, bioavailable testosterone

## Abstract

**INTRODUCTION:**

Water-pipe (WP) smoking is the most common method of tobacco consumption in the Middle-East and is rapidly spreading on a global scale. Although, water-pipe smoking is linked to various diseases, such as emphysema and various types of cancers, its effect on testosterone levels has yet to be investigated. This study explores the effect of water-pipe smoking on serum testosterone levels in males in Qatar.

**METHODS:**

In this cross-sectional sample within a cohort study, we retrieved data for a total of 1000 male volunteers from the Qatar BioBank (QBB) project. A self-reported questionnaire was used to determine the water-pipe smoking status of participants. Moreover, participants were stratified based on the frequency of smoking. Total testosterone and sex hormone binding globulin (SHBG) were measured clinically, whereas free testosterone and bioavailable testosterone were calculated using Vermeulen’s equation. Hormone values of 541 males (277 water-pipe smokers and 264 non-smokers) were compared using multiple regression analysis based on water-pipe smoking status after adjusting for confounding factors.

**RESULTS:**

No statistically significant difference was observed between WP smokers and non-water-pipe smokers in the likelihood of having lower or higher total testosterone, after adjustment for confounding factors. Similar results were found in free testosterone, bioavailable testosterone, and sex hormone binding globulin (all p>0.05). When compared with the reference group, both light and heavy water-pipe smokers had a similar likelihood of circulating low total testosterone levels (OR=0.83, 95% CI: 0.46–1.49; and OR=0.80, 95% CI: 0.43–1.49; respectively).

**CONCLUSIONS:**

Our results reveal, for the first time, that there is no significant change in total testosterone, free testosterone, bioavailable testosterone and sex hormone binding globulin in waterpipe smokers compared to non-water-pipe smokers. Therefore, we believe that further studies are needed to confirm the effect of water-pipe smoking on testosterone in different populations.

## INTRODUCTION

Tobacco smoking is considered responsible for numerous serious human diseases, according to the World Health Organization (WHO), making it one of the main causes of preventable illness and mortality worldwide. Tobacco smoking is consumed in various forms encompassing cigarette, cigar, e-cigarette, and water-pipe (WP). Today, WP smoking is the most widespread method of tobacco consumption in the Middle-East and is rapidly spreading globally as a form of social conduct^1^. WP and cigarettes both contain similar toxins such as nicotine, carbon monoxide and polycyclic aromatic hydrocarbons (PAH)^2^; hence, it is a misconception that WP smoking is less harmful than cigarette smoking. More specifically, WP smoke contains significantly more carbon monoxide, equivalent to that from 10 cigarettes per day^3^. In addition to these toxins, WP smoking also exposes smokers to fine charcoal particles, resin particles, flavourings, sweeteners, and added perfumes^4^.

Furthermore, recent studies clearly show the harmful effects of WP smoking on human health, which include chronic bronchitis, emphysema, coronary artery disease, lung, gastric and esophageal cancers, and osteoporosis along with significant effects on embryogenesis^5,6^. Meanwhile, earlier studies assessed the relationship between cigarette smoking and testosterone levels in men arriving at controversial conclusions, with undetermined mechanisms^7,8^.

While there is no investigation concerning the effect of WP smoking on the serum levels of testosterone in men, a study on male mice showed that chronic WP smoke exposure has adverse effects on the male reproductive system including testosterone levels^9^. The mechanism by which WP smoking could affect testosterone levels is under research^9^. Also, changes in testosterone levels have been linked to many disorders such as erectile dysfunction, cardiovascular diseases, Type-2 diabetes mellitus (DM), metabolic syndrome, and obesity^10^. Therefore, evaluating the outcome of WP smoking on testosterone levels is essential to identify and/or explain known WP smoking-associated pathologies. This study aims to explore the association between WP smoke and serum levels of total testosterone (TT), free testosterone (FT), bioavailable testosterone (BioT) and sex hormone binding globulin (SHBG) among men in Qatar. Thus, this investigation addresses, for the first time, an important gap in the outcome of WP smoking on testosterone levels.

## METHODS

### Study design and population

Our study was conducted using data from the Qatar BioBank (QBB) project (https://www.qatarbiobank.org.qa). In brief, QBB is a population-based longitudinal cohort that recruits adults who are nationals and long-term (>15 years) residents of Qatar between 18 and 89 years of age, and follows them up to record their health status. Recruitment started in December 2012 and collected information of 12000 participants, and continues to date. Participation was voluntary, and bookings were made through the website or by phone calls. After obtaining a written informed consent, all participants answered a self-administered questionnaire. Also, anthropometrics and body composition were measured, followed by a collection of blood and urine samples.

Our cross-sectional sampling within the cohort study^11^ collected a random sample of 1000 males from the 5500 adult males who participated in QBB until December 2017 and measured the association between WP smoking and testosterone level. Up to the time the study was conducted, the study group had been exposed to WP smoking for a mean duration of 13 years. Exclusion criteria for our study included history of DM, liver cirrhosis, psychiatric disorders, neoplastic conditions, pelvic surgeries, obesity stage II or III, body mass index (BMI > 35), participants who smoked WP before but were no longer smokers at the time of the study, and participants with missing data of TT, SHBG, or WP smoking status. The final sample size was 541 participants for whom we could detect a difference of 1.5 units in means or 10% in proportion between exposed and non-exposed groups with a power of 80% and an alpha level of 0.05. This study was approved by the QBB institutional review board (IRB) and Qatar University IRB, where the study was conducted.

### Confounding factors

**Figure 1:**
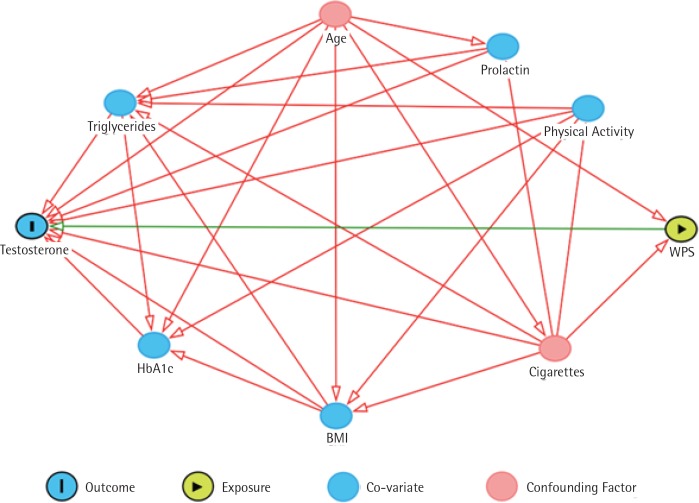
A directed acyclic graph that shows the relationship between testosterone levels as a dependent variable, and WPS (water-pipe smoking) as the independent variable in the presence of several factors which are age, cigarette smoking, triglycerides, HbA1c, BMI, Prolactin, and physical activity. The figure shows that only age and cigarette smoking are confounding factors

Factors that are associated with testosterone levels were investigated as potential confounding factors. To assess which of these factors can be considered true confounding factors and should be included in the analysis, a directed acyclic graph (DAG)^12^ was developed as illustrated in Figure 1. The DAG included the exposure, outcome and other factors that have a relationship with the outcome, such as age, concurrent cigarette smoking, BMI, triglycerides, HbA1c, level of physical activity, and prolactin level. Waist-hip ratio was not included as it has a linear relationship with BMI. The DAG revealed that only age and cigarette smoking status are confounding factors that are associated with both exposure and outcome, thus, these have been adjusted for in the analysis (Figure 1). Alcohol consumption was not included as it is exceedingly rare in Qatar.

### WP smoking status

Participants answered a self-administered questionnaire about their WP smoking status, the frequency of smoking, and duration of smoking. Men who smoked at least once in the last month were considered smokers, men who had never smoked throughout their life were considered non-smokers, and men who stopped smoking were excluded from the analysis to avoid misclassification bias. Smokers were stratified into heavy and light smokers; heavy smokers were classified as men who smoked more than once per week, light smokers were classified as men who smoked three times or less in the last month. Heavy and light smokers had smoked for 11.9 and 13.7 years, respectively, which were considered similar (p=0.084). Therefore, smoking duration was not considered when smokers were stratified.

### Anthropometrics and laboratory measurements

Anthropometrics were measured by a trained nurse. TANITA BC-418 MA instrument was used to measure participants’ weight (Kg) and height (m). BMI was calculated and treated as a continuous variable.

Physical activity data were collected by a questionnaire. The patients were asked about the total number of days and the average time spent per day in various activities, used to calculate the total metabolic equivalence^13^. Cigarette smoking status was self-reported by the participants and then categorised to non-smokers, light smokers, and heavy smokers based on the frequency and number of cigarettes smoked per day.

Blood samples were collected from patients in the morning (7:30–13:30) and immediately transferred frozen (at -80°C) to Hamad Medical Corporation’s (HMC) chemistry laboratory, Doha, Qatar. The samples were analysed within 2 hours following their standard operating procedures. TT was measured by the Abbott testosterone method following the manufacturer’s specifications. SHBG was measured by immunometric assay with fluorescence detection on the DPC Immulite 2000 analyzer following the manufacturer’s protocol. FT and BioT were calculated using Vermeulen’s equation^14^. Triglycerides were measured by GPO-PAP. HbA1c was measured by turbidimetric inhibition immunoassay (TINIA).

### Statistical analysis

Normal distribution was assessed by Shapiro-Wilk test and histograms. Means of normally distributed data were compared using t-test while Mann Whitney U-test was used for non-normally distributed data. Categorical variables were compared using chi-squared test. Hormone levels were categorised into tertiles rather than clinical cutoffs as they do not apply to our population^15^. To test whether missing values were missing completely at random (MCAR), Little’s MCAR test with expectation maximisation algorithm was conducted. Hormones categories were included as the dependent variables in a multiple regression analysis to test the relationship with WP smoking level (independent variable) after adjustment for age and cigarette smoking. The lower and higher tertiles of hormone levels were compared with the middle tertile to test whether WP smoking increases or decreases these hormones. Odds ratios (OR) and associated 95% confidence intervals (CI) were obtained. Statistical Package for Social Sciences (SPSS) 64-bit version 23 was used to carry out the previous tests. All tests were two-tailed, and results were considered statistically significant if p-values were less than 0.05.

## RESULTS

**Figure 2:**
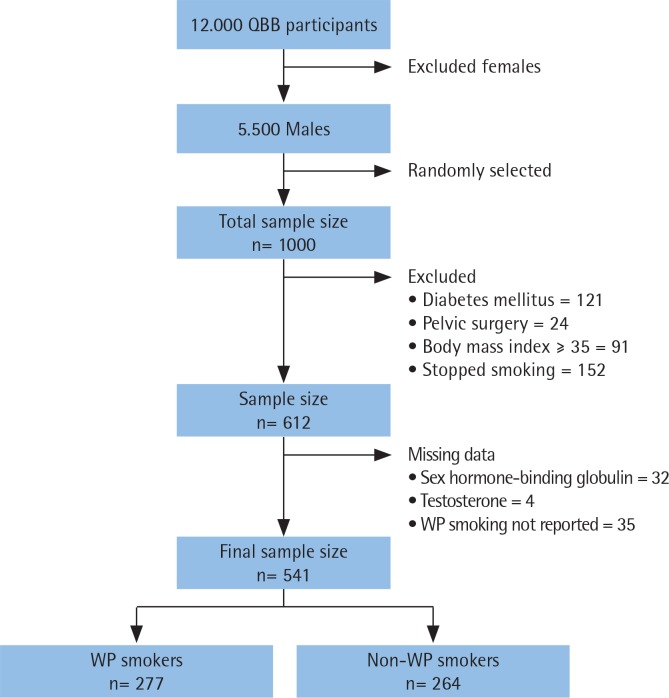
A flowchart that shows the study population and the final sample size (QBB: Qatar BioBank, WP: water-pipe)

A total of 1000 participant were randomly selected from the 5500 males who attended the QBB project. Of these, 388 participants were excluded for not meeting the eligibility criteria, and 71 were excluded for missing values. Little’s MCAR test showed that missing values were completely at random (p=0.734). The analyses were conducted on the remaining 541 participants; 277 subjects (51%) were WP smokers and 264 (49%) non-WP smokers (Figure 2).

The characteristics of the sample population by WP smoking status are defined by mean ± SD, as depicted in Table 1. Of the eligible participants, 96% were Qatari males. Thus, the impact of ethnicity is insignificant. Participants in our sample were in the age range 19–80 years with mean age among non-smokers higher than WP smokers (39.0±11.4 and 34.2±8.6, respectively; p<0.001). The mean duration of WP smoking status was previously addressed, and no significance was detected in light smokers compared to heavy smokers (p=0.084). Moreover, the mean±SD for TT was 19.4±7.7 nmol/L, for FT 0.4±0.2 nmol/L, and for BioT 9.9±3.8 nmol/L, all significantly higher in WP smokers than in non-WP smokers (p=0.01); nevertheless, these results were not adjusted for other confounding factors, as mentioned in the Methods section. Also, HBA1c was found to be significantly higher in non-WP smokers compared to WP smokers (p=0.008). On the other hand, no significant difference was detected in the mean values of BMI (p=0.71), SHBG (p=0.97), physical activity (p=0.89), and triglycerides (p=0.51) between WP smokers and non-WP smokers. WP smokers are more likely to smoke cigarettes compared to non-WP smokers (p<0.001).

**Table 1:** Baseline characteristics of 541 subjects grouped by WP smoking status

	*WPS n=277*	*NS n=264*	*p*
**Age (years)**	34.2±8.6	38.9±11.4	<0.001
**Age (years)BMI (kg/m^2^)**	27.0±3.9	27.2±3.9	0.71
**Physical activity (mL^2^/kg Age (years)/min)**	33.0±79.0	26.7±53.2	0.90
**Cigarette smoking status**			
Non-smokers	98 (35)	246 (93)	<0.001
Light-smokers	71 (26)	2 (1)	
Heavy-smokers	108 (39)	16 (6)	
**HBA1c (%)**	5.3±0.6	5.4±0.5	0.008
**Triglycerides (mmol/L)**	1.5±1.0	1.6±1.1	0.51
**TT (nmol/L)**	19.4±7.7	17.9±6.8	0.01
**FT (nmol/L)**	0.4±0.2	0.3±0.1	0.01
**BioT (nmol/L)**	9.9±3.8	9.1±3.1	0.01
**SHBG (nmol/L)**	34.4±16.5	34.6±15.5	0.96

Data are expressed as mean±standard deviation or as number of subjects and percentage, n (%). The p-values were obtained by t-test or Mann-Whitney U-test. WPS: water-pipe smoking, NS: non-smoker, BMI: body mass index, TT: total testosterone, FT: free testosterone, BioT: bioavailable testosterone, SHBG: sex hormone binding globulin.

Table 2 illustrates the association between WP smoking and testosterone levels while adjusting for age and number of cigarettes smoked. TT levels were stratified into tertiles; low (1.0–14.9), middle (15.0–20.6), and high (20.7–51.3) nmol/L. The middle tertile values for the rest of the hormones were defined as 0.314–0.413 nmol/L for FT, 7.9–10.5 nmol/L for BioT and 26.4–37.9 nmol/L for SHBG. Our analysis showed that WP smoking neither increases nor decreases TT, FT, BioT, or SHBG, when adjusted for potential confounders. Compared with the reference group, both light and heavy WP smokers had a similar likelihood of circulating low TT levels (OR=0.83, 95% CI: 0.46–1.49; and OR=0.80, 95% CI: 0.43–1.49; respectively). Likewise, both light and heavy WP smokers had an insignificant statistical difference on the likelihood of circulating high TT levels (OR=0.92, 95% CI: 0.52–1.64; and OR=0.71, 95% CI: 0.38–1.33; respectively). Similar findings were observed for other sex hormones as shown in Table 2.

**Table 2:** Adjusted odds ratios and 95% confidence intervals of hormone levels divided into tertiles (Low and High, as defined) according to the level of WP smoking

*Tertiles*	*TT*		*FT*		*BioT*		*SHBG*	
*WPS Status*								
	Low (1.0–14.9)	High (20.7–51.3)	Low (0.04–0.3)	High (0.4–1.4)	Low (0.9–7.9)	High (10.6–31)	Low (2.5–26.0)	High (38.0–118.0)
Light	0.83 (0.46–1.49)	0.92 (0.52–1.64)	0.84 (0.46–1.51)	0.79 (0.44–1.42)	0.80 (0.44–1.45)	0.73 (0.40–1.31)	0.75 (0.42–1.34)	0.76 0.42–1.38)
Heavy	0.80 (0.43–1.49)	0.71 (0.38–1.33)	0.88 (0.47–1.64)	0.68 (0.36–1.27)	0.80 (0.42–1.50)	0.71 (0.38–1.34)	0.94 (0.50–1.77)	1.08 (0.57–2.03)

Results were obtained by multiple logistic regression after adjustment for age and cigarette smoking. Abbreviations as in Table 1 footnote.

## DISCUSSION

This is the first study exploring the relationship between WP smoking and serum testosterone values. There are numerous studies that have been conducted evaluating the impact of cigarette smoking on testosterone serum values^7-9,16-20^. Further, studies have shown that WP smoke contains diverse toxic compounds and highly concentrated nicotine and tobacco intoxicants compared to cigarettes^2^. Moreover, the carcinogenic PAH accumulated in one WP smoking session is 20 times greater than the PAH produced from a single cigarette^21^. Also, studies that explore the consequences of WP smoking on human health are scarce. Thus, due to the limited data on WP smoking and associated injurious effects, this study aimed to assess the relationship between WP smoking and testosterone levels in men in Qatar. Our results reveal no association between WP smoking and serum testosterone values, after adjusting for age and cigarette status.

Although the effects of tobacco smoking on TT and FT values are controversial, the present study is supported by several other investigations^19,20,22^. For example, according to a population-based study of 890 male participants of age 22–90 years, no significant effect of smoking on TT and FT values was detected, after adjusting for alcohol consumption, stroke, DM, and coronary heart disease as confounders^18^. Moreover, 255 men of age 30–70 years were studied in a cross-sectional investigation and no significant effect of smoking was found on TT and FT values, after adjusting for age, BMI, WHR, and prolactin^17^. Although these studies adjusted for non-confounding factors that should not be adjusted, such as BMI, WHR and prolactin, the findings of these studies are consistent with our results^17,18^.

On the other hand, there are other investigations that report increases in the TT values associated with smoking^7,8,16^. In this regard, 1150 male patients were analyzed in an infertility unit for sexual dysfunction and a significant increase in TT serum levels in smokers compared to non-smokers was noticed, after adjusting for age^16^. Nevertheless, the participants included in this study were referrals for sexual dysfunction, which introduces selection bias and decreases its generalizability. Therefore, the validity, as well as the suitable application to fit male smokers, is debatable. Moreover, in a cross-sectional study, 3427 male participants were investigated to assess smoking on TT levels and an increase in TT levels in smokers compared to non-smokers was found, adjusted for age^7^. In contrast, studies suggest that smoking decreases TT levels; a case-control study involving 45 men, age 25–35 years, searched for an association between cigarette smoking and endocrine profile and found a decrease in TT values^23^. This study was also supported by another study^24^.

Furthermore, our results reveal that neither WP smoke nor cigarettes have an impact on the BioT in smokers compared to non-smokers. As such, our data agree with the limited studies published on this topic to date; English et al.^25^ and Halmenschlager et al.^17^ both examined smoking on BioT levels and found no significant effect, after adjustment for age as a confounder.

In general, most of the testosterone found in the human body is bound to SHBG. It is believed that 65–80% of the TT is bound to SHBG^26^. Therefore, variations in SHBG could proportionally indicate changes in TT values. Thus, few studies suggest that smokers have a higher SHBG level compared to non-smokers^25,27^. Here, we reveal that there is no significant difference in SHBG levels between WP smokers and non-WP smokers after adjusting for confounders, and this is supported by other investigations^17,20,28^.

Several studies in the literature report that WP smoking plays a mechanistic role in affecting testosterone levels^23,29-33^. These mechanisms are ambiguous; thus, theories so far are controversial, linking tobacco smoke with the outcome of testosterone values to several different pathways^23,29-32^. For instance, Kimura et al.^29^ anticipated that nicotine causes gamma-aminobutyric acid to be released which then inhibits gonadotropin-releasing hormone via the hypothalamus-pituitary pathway. In contrast, studies also proposed theories suggesting tobacco smoking could increase testosterone values^31^. For example, Wu et al.^31^, documented that the compensatory increase in luteinizing hormone (LH) primarily increases SHBG which would, therefore, increase total testosterone. However, our study does not agree with the above theories. Consequently, we propose that these effects do not induce a clinically significant alteration in testosterone values.

Although the present study did not detect a significant effect on androgen values in WP smokers when compared to non-WP smokers, an impact of tobacco smoking on the reproductive system cannot be ruled out. There are numerous studies in the literature that suggest tobacco smoking decreases reproduction^23,33^. It is reported that nicotine and its substrate, cotinine, decreases the semen parameters and results in infertility^34^. Also, Gornig and Schirren^35^, demonstrated that smokers have a significant decrease in sperm motility and count. Their results were supported by several other studies^23,36,37^. These studies indicate that the decrease in semen parameters could be interpreted by the abundance of nicotine and lead, which are delivered through WP smoking. Thus, we believe that further studies are needed to confirm these data in other populations and to explore the effect of WP smoking on the human reproductive system as a whole.

### Limitations and strengths

There are several strengths and limitations in our study that need to be highlighted. First, the strengths include that our sample was selected randomly from QBB where subjects’ history of exposures were collected, and all tests were performed in a single laboratory. Second, the sample consisted of volunteers that were not referred by healthcare providers. Third, we avoided adjustment for non-confounding factors, which can introduce selection bias, by performing a DAG. On the other hand, the main limitation of our study was that we calculated FT and BioT using Vermeulen’s equation^14^ as they were not measured clinically by QBB. Although the calculated FT is a reliable index of the measured FT, it is recommended that testosterone values are measured using liquid chromatography-tandem mass spectroscopy as it is the gold standard, rather than calculating it, because of uncertainty^38^. Nevertheless, measuring BioT would be expensive and impractical. Another limitation was that we had only a single measurement for the hormone values used in the study. Also, the blood samples were collected within a wide time interval. Thus, changes in testosterone levels may be masked by circadian rhythm.

Finally, it is worth to mention that alcohol consumption and medications were not taken into account because of unavailable data. However, alcohol consumption is extremely rare in Qatar due to religious and social reasons and would not have affected the analysis.

## CONCLUSIONS

This study investigated for the first time the association between WP smoking and testosterone values. Our results demonstrate that there is no significant change in TT, FT, BioT and SHBG in WP smokers compared to non-WP smokers, after adjusting for age and cigarette smoking as confounders.
